# An algorithm to identify functional groups in organic molecules

**DOI:** 10.1186/s13321-017-0225-z

**Published:** 2017-06-07

**Authors:** Peter Ertl

**Affiliations:** Novartis Institutes for BioMedical Research, 4056 Basel, Switzerland

**Keywords:** Functional group, Chemical functionality, Organic chemistry, Medicinal chemistry

## Abstract

**Background:**

The concept of functional groups forms a basis of organic chemistry, medicinal chemistry, toxicity assessment, spectroscopy and also chemical nomenclature. All current software systems to identify functional groups are based on a predefined list of substructures. We are not aware of any program that can identify all functional groups in a molecule automatically. The algorithm presented in this article is an attempt to solve this scientific challenge.

**Results:**

An algorithm to identify functional groups in a molecule based on iterative marching through its atoms is described. The procedure is illustrated by extracting functional groups from the bioactive portion of the ChEMBL database, resulting in identification of 3080 unique functional groups.

**Conclusions:**

A new algorithm to identify all functional groups in organic molecules is presented. The algorithm is relatively simple and full details with examples are provided, therefore implementation in any cheminformatics toolkit should be relatively easy. The new method allows the analysis of functional groups in large chemical databases in a way that was not possible using previous approaches.

**Electronic supplementary material:**

The online version of this article (doi:10.1186/s13321-017-0225-z) contains supplementary material, which is available to authorized users.

**Graphical abstract:**
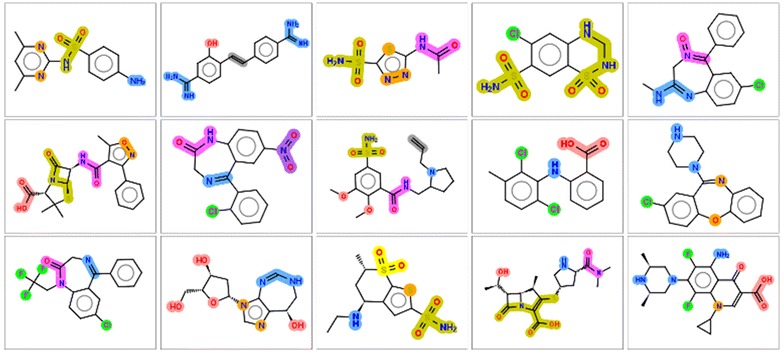
.

## Background

The concept of functional groups (FGs)—sets of connected atoms that determine properties and reactivity of parent molecule, forms a cornerstone of organic chemistry, medicinal chemistry, toxicity assessment, spectroscopy and, last but not least, also chemical nomenclature. The study of common FGs forms substantial part of basic organic chemistry curriculum. Numerous scientific papers and books focus on properties and reactivity of various FGs. A well known example is the classical book series “Chemistry of functional groups” describing various classes of organic molecules [1] consisting of over 100 volumes. There is, however, surprisingly little attention paid to the study of functional groups from the cheminformatics point of view. The majority of theoretical studies are utilizing FGs as a basis of chemical ontologies, where FGs are “keys” that are used to hierarchically classify molecules into categories [2]. An example of this type of publications is work by Bobach et al. describing a rule-based definition of chemical classes to classify compounds into classes [3] or the ClassyFire software [4] developed in the Wishart’s group allowing chemists to perform large-scale automated chemical classification based on a structure-based chemical taxonomy consisting of over 4800 categories.

Various substructure features are often used in cheminformatics in connection with machine learning to develop models to predict biological activity or properties of molecules [5]. In this approach the substructure descriptors are generated by extracting groups of atoms from a molecule using a predefined algorithm. Examples of such descriptors are linear or atom centered fragments, topological torsions, pharmacophoric triplets and many others. Although such fragment descriptors are very useful, they do not provide description of functional groups. The fragments are generally strongly overlapping and are generated for all parts of a molecule without considering their potential chemical role.

One of the first software tools to identify FGs was the checkmol program written by Haider [6] that was able to identify about 200 FGs. Recently an extended version of the program containing 583 manually curated functionalities encoded as SMARTS was published [7]. This list includes also numerous heterocyclic rings and general structural patterns (i.e. 5-membered aromatic ring with 1 heteroatom). These substructure features are used to develop QSAR models for prediction of toxicity and various molecular physicochemical properties. The well-known ZINC database and related web-based software suite [8] stores about 500, so called, chemical patterns, that speed-up substructure searches and allow estimation of molecule reactivity. The patterns include PAINS filters [9] that identify frequent hitters interfering with biochemical screens as well as some other substructures. Another widely used set of substructures used to identify potentially reactive or promiscuous molecule has been defined by Eli Lilly scientists based on their experience with internal screening campaigns [10]. Recently a set of generic chemical functionalities called ToxPrint chemotypes that describe molecule substructure and reaction features and atom and bond properties was defined within the ToxPrint program [11]. The main goal of the tool is to use these features in toxicity modelling.

We are not aware of any software system able to identify FGs that is not based on manually curated set of substructure features, but instead automatically identifies all functional groups in a molecule. The algorithm presented in this article is an attempt to solve this scientific challenge.

## Methods

### Identification and extraction of functional groups

The majority of FGs contain heteroatoms. Therefore our approach is based on processing heteroatoms and their environment with the addition of some other functionalities, like multiple carbon–carbon bonds.

The algorithm is outlined below:

1. mark all heteroatoms in a molecule, including halogens
2. mark also the following carbon atoms:
  atoms connected by non-aromatic double or triple bond to any heteroatom
  atoms in nonaromatic carbon–carbon double or triple bonds
  acetal carbons, i.e. sp3 carbons connected to two or more oxygens, nitrogens or sulfurs; these O, N or S atoms must have only single bonds
  all atoms in oxirane, aziridine and thiirane rings (such rings are traditionally considered to be functional groups due to their high reactivity).
3. merge all connected marked atoms to a single FG
4. extract FGs also with connected unmarked carbon atoms, these carbon atoms are not part of the FG itself, but form its environment.

The algorithm described above iterates only through non-aromatic atoms. Aromatic heteroatoms are collected as single atoms, not as part of a larger system. They are extended to a larger FG only when there is an aliphatic functionality connected (for example an acyl group connected to a pyrrole nitrogen). Heteroatoms in heterocycles are traditionally not considered to be “classical” FGs by themselves but simply to be part of the whole heterocyclic ring. The rationale for such treatment is enormous diversity of heterocyclic systems. For example in our previous study [12] nearly 600,000 different heterocycles consisting of 1–3 fused 5- and 6- membered rings were enumerated.

After marking all atoms that are part of FGs as described above, the identified FGs are extracted together also with their environment—i.e. connected carbon atoms, when the type of carbon (aliphatic or aromatic) is also preserved.

We do not claim that this algorithm provides an ultimate definition of FGs. Every medicinal chemist has probably a slightly different understanding about what a FG is. In particular the definition of activated sp3 carbons may create some discussion. In the present algorithm we restricted our definition only to classical acetal, thioacetal or aminal centers (i.e. sp3 carbons having at least 2 oxygens, sulfurs or nitrogens as neighbors) and did not consider other similar systems, i.e. alpha-substituted carbonyls or carbons connected to S=O or similar bonds. During the program development phase various such options have been tested, and this “strict” definition provided the most satisfactory results. Extension of FGs also to alpha-substituted carbonyls (i.e. heteroatom or halogen in alpha position to carbonyl) and similar systems more than triple the number of FGs identified, generating many large and rare FGs. Since our major interest was in comparing various molecular datasets and not in reactivity estimation we implemented this strict definition of acetal carbons. To assess the possible reactivity of molecules, various substructures filters are available, as for example already mentioned PAINS [9] or Eli Lilly rules [10].

**Fig. 1:**
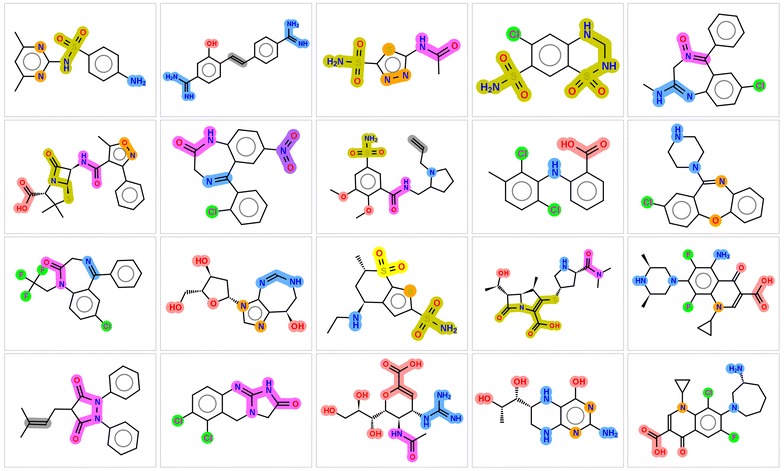
Example of functional groups identified. Groups are color coded according to their type

To illustrate better the algorithm some examples of FGs identified for few simple molecules are shown in Fig. 1.

### Generalization of functional groups

**Fig. 2:**
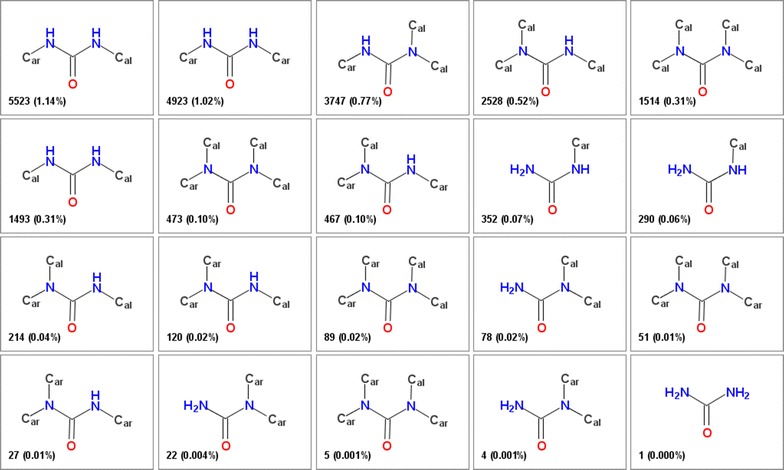
Various forms of the urea functionality differing in the environment patterns. The *numbers* in the corner indicate the number of molecules in ChEMBL in which this particular group is present and the percentage

FGs, particularly those with several connection points, may be present in numerous forms differing by variation in their environment. The attachment points may be unsubstituted (i.e. the valences are filled by hydrogens) or connected to aliphatic or aromatic carbons with large number of possible combinations. A simple amide group with 3 connection points may form 18 such variations (two connections on nitrogen are considered to be symmetrical here). As another example list of 20 ureas with different environments extracted from the ChEMBL database (vide infra) is shown on Fig. 2. For the more complex groups the number of possible variations is even considerably larger.

In most cases, however, it is not necessary to go into such level of detail. When studying frequency statistics of FGs in chemical databases one is usually interested in percentage of molecules with, say, urea or sulfonamide functionalities and not in the environment details. It would be therefore desirable to merge FGs based on the important “central” moiety. One needs to be careful here, however. In some special cases, particularly for smaller FGs the differences in the environment are very important, for example to distinguish between alcohols and phenols or amines and anilines. To consider these different scenarios the generalization scheme described below was developed:

1. environments on carbon atoms are deleted, the only exception are substituents on carbonyl that are retained (to distinguish between aldehydes and ketones)
2. all free valences on heteroatoms are filled by the “R atoms” (this atom may represent hydrogen or carbon) with exception of:
  hydrogens on the –OH groups
  hydrogens on the simple amines and thiols (i.e. FGs with just single central N or S atom) are not replaced, this allows to distinguish secondary and tertiary amines, and thiols and sulfides.
3. all remaining environment carbons (on heteroatoms and carbonyls) are replaced by the “R atoms”; exceptions are environments on single atomic N or O FGs with one carbon connected, where this carbon is retained also with its type (aliphatic or aromatic), this allows to distinguish between amines and anilines, and alcohols and phenols.

This scheme provides a good balance between preserving sufficient, chemically meaningful details on one side and generalization on the other side. Examples of generalized FGs created by this procedure are shown in the following section.

## Results and discussion

The algorithm outlined above was implemented using the Novartis in-house cheminformatics system written in Java and was tested by identifying and collecting FGs for bioactive portion of the ChEMBL database [13], consisting of ~483,000 molecules with activity below 10 um on any ChEMBL target. Organometallic structures were discarded and all molecules were standardized by removing counterions and neutralizing atomic charges.

**Fig. 3:**
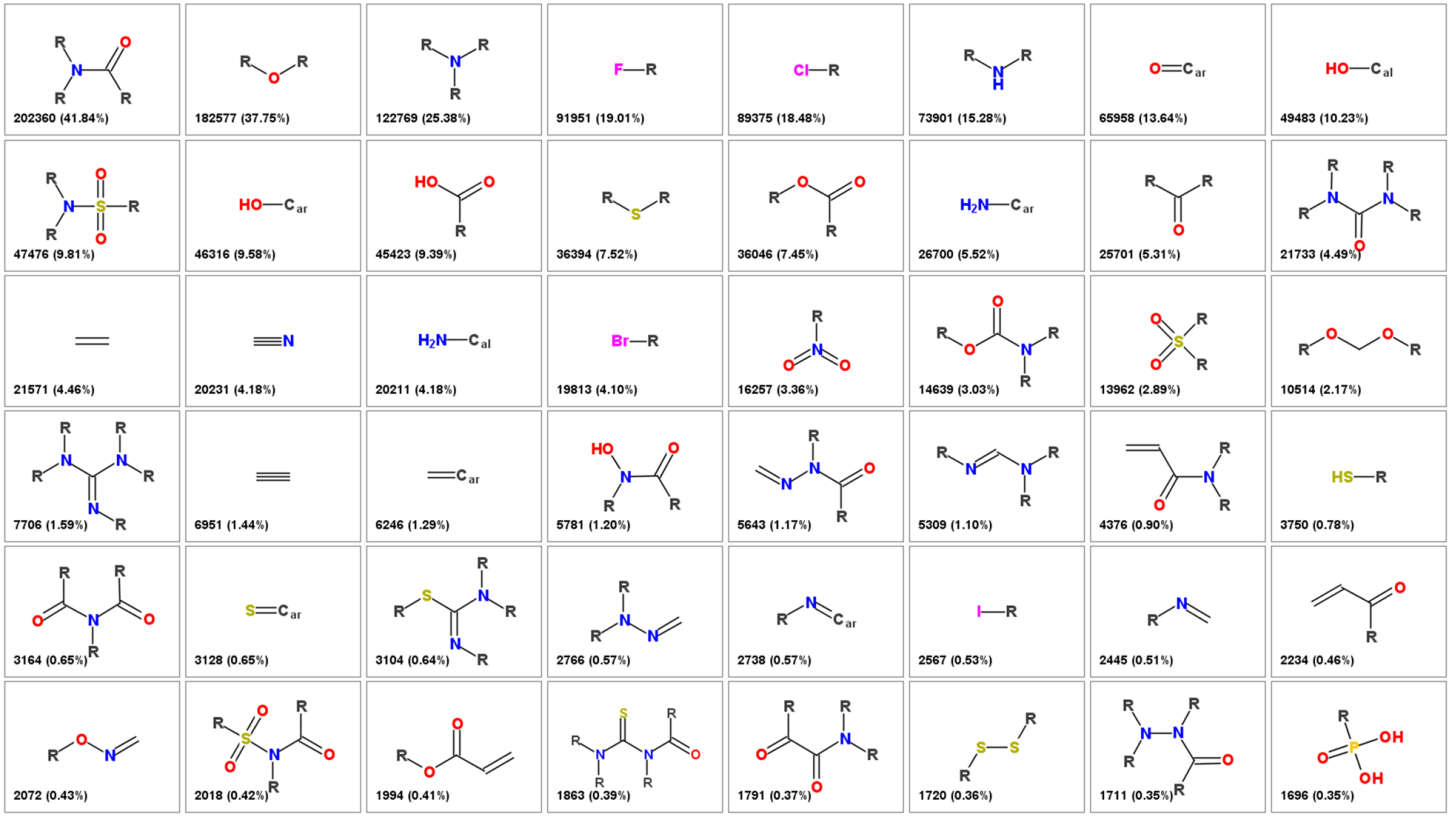
The most common functional groups from the ChEMBL database. The *numbers* in the corner indicate the number of molecules in ChEMBL in which this particular group is present and the percentage

The most frequent FGs identified (not counting aromatic heteroatoms) are shown in Fig. 3. The most common FG is amide (present in 41.8% of molecules), followed by the ester group (37.8%), tertiary amine (25.4%) and fluoro (19.0%) and chloro (18.5%) substituents. Altogether our procedure identified 3080 FGs. Their distribution is a typical power law “long tail” distribution (similarly to the distribution of other substructure features like substituents or linkers [14]) with few very common groups and large number of infrequent groups. 33 FGs are present in more than 1% of molecules, 88 in more than 0.1% of molecules. 1218 groups (39.5%) are singletons (present in only one molecule). A list of 768 FGs that are present in at least 10 ChEMBL molecules is provided in pseudo-SMILES notation as Additional file 1.

**Fig. 4:**
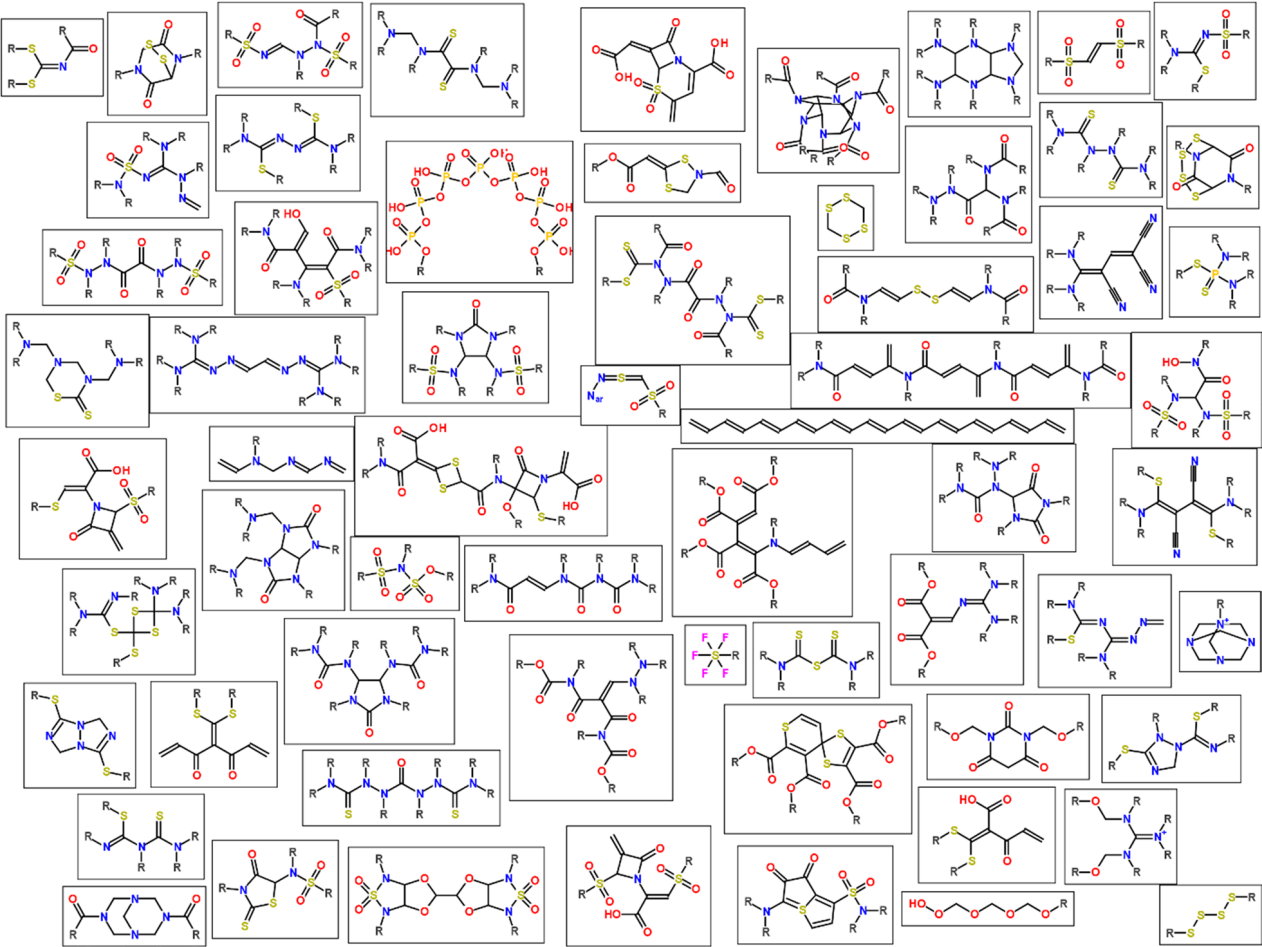
Example of some exotic functional groups identified displayed as molecule cloud [15]

To illustrate diversity of FGs a selection of some more exotic functionalities is shown in Fig. 4. This shows that when analyzing functionalities in large chemical databases it is not sufficient to limit ourselves to a predefined list of patterns, but it is necessary to identify all FGs.

To validate this new approach a comparison with the current state-of-the-art program for identification of FGs—checkmol [6] was performed. Checkmol analyses molecules and identifies FGs from a predefined set of 204 functionalities. The philosophy of checkmol is slightly different from that of our approach, checkmol defines FGs in a hierarchical manner so for comparison we used the most detailed substructure defined in checkmol. In the ChEMBL dataset checkmol identified 165 FGs. Comparison of the results, i.e. the percentage of occurrences of the most common FGs (34 groups present in at least 2% of ChEMBL molecules) is shown in Table 1. The results show very good agreement of the two methods. The differences are in most cases within tenth of percent. For the simple aromatic substituents checkmol identifies generally slightly less FGs than our approach (for example arylchloride 18.13 vs 18.17% or phenol 9.52 vs 9.58%) what is caused probably by slightly different definitions of aromaticity by both systems. The largest differences between the 2 approaches may be observed for simple functionalities that are often part of the more complex FGs. The most prominent example is alkene with checkmol result 8.63% while our method provides only 4.46%. The reason for this is the fact that the alkene is often part of relatively common more complex FGs (for example acrylamide, acryloyl etc.) that are not recognized by checkmol but are identified by our algorithm as FGs on their own. Another such example is ketone (7.82% by checkmol, 5.31% by our approach) when the acyl functionality is also often part of more complex FGs, the acylhydrazone itself accounting for 1.17% of the difference. The most frequent FGs that are not included in the checkmol list that are identified by our algorithm are acylhydrazone (C–C(=O)–N–N=C, present in 1.17% of ChEMBL structures), acrylamide (C=C–C(=O)–N, 0.90%), acryloyl (C=C–C(=O)–C, 0.46%), acylsulfonamide (C–C(=O)–N–S(=O)=O, 0.42%) and acrylester (C=C–C(=O)–O–C, 0.41%).

**Table 1:** Comparison of frequency (in %) of FGs identified by checkmol [6] and by the presented algorithm

Functional group	Checkmol	This study	Functional group	Checkmol	This study
Secondary amide^a^	33.34	33.21	Lactam	6.16	4.65
Alkyl aryl ether	28.27	28.93	Urea	5.94	4.49
Aryl chloride	18.13	18.17	Sec. aliphatic/aromatic amine	5.90	6.08
Tert. aliphatic amine	17.89	17.90	Sec. aliphatic amine	5.72	5.77
Tertiary amide^a^	15.99	14.75	Prim. aromatic amine	5.50	5.52
Aryl fluoride	12.37	12.38	Carbonitrile	4.84	4.18
Oxoarene	11.61	13.64	Hydrazine derivative	4.45	4.32
Alcohol	11.64	10.23	Sec. aromatic amine	4.34	4.41
Sulfonamide	11.28	9.81	Prim. aliphatic amine	4.14	4.18
Tert. aliphatic/aromatic amine	10.90	10.96	Aryl bromide	4.02	4.03
Carboxylic acid	9.93	9.39	Primary amide	3.48	3.36
Phenol/hydroxyarene	9.52	9.58	Nitro compound	3.40	3.36
Dialkyl ether	8.65	9.42	Urethane	3.33	3.03
Alkene	8.63	4.46	Sulfone	2.98	2.89
Carboxlic acid ester	8.47	7.45	Diaryl ether	2.87	2.81
Ketone	7.82	5.31	Acetal	2.26	2.17
Thioether	7.51	7.52	Guanidine	2.21	1.59

^a^Including lactames

In summary, the agreement between the both approaches is very good. This shows that systems based on a well selected list of substructures (as apparently checkmol is) can provide useful information about the FG composition of general molecular datasets. The advantage of our algorithm of automatic identification of FGs is its ability to handle more complex FGs that are often present in specialized collections of molecules. Examples identified by this simple analysis are activated alkenes (that act as a Michael acceptors and are often used as covalent binders). Another advantage of the algorithmic approach over predefined set of substructures is more fine graining of the results (like the detailed classification of ureas mentioned previously, that may be important for analyzing properties like solubility) and the easy possibility to fine-tune the algorithm to fit the needs of a particular analysis (for example library diversity or QSAR studies).

## Conclusions

A new algorithm to identify all functional groups in organic molecules is presented. The algorithm is relatively simple and full details with examples are provided, therefore implementation in any cheminformatics toolkit should be relatively easy. The author is willing to provide help in any such endeavor. The new method allows to analyze FGs in large chemical databases in a way that was not possible using previous approaches. Several such studies focusing on differences in distribution of FGs between bioactive and “average” molecules as well as identification of functionalities typical for different classes of natural products are underway.

## Additional file

**Additional file 1.** List of 768 functional group with their frequencies in pseudo-SMILES notation.

## References

[1] Patai’s Chemistry of Functional Groups. Wiley. http://onlinelibrary.wiley.com/book/10.1002/9780470682531

[2] Feldman HJ, Dumontier M, Ling S, Haider N, Hogue CW (2005). CO: a chemical ontology for identification of functional groups and semantic comparison of small molecules. FEBS Lett.

[3] Bobach C, Böhme T, Laube U, Püschel A, Weber L (2012). Automated compound classification using a chemical ontology. J Cheminform.

[4] Djoumbou Feunang Y, Eisner R, Knox C, Chepelev L, Hastings J, Owen G, Fahy E, Steinbeck C, Subramanian S, Bolton E, Greiner R, Wishart DS (2016). ClassyFire: automated chemical classification with a comprehensive, computable taxonomy. J Cheminform.

[5] Lewis RA, Wood D (2015). Modern 2D QSAR for drug discovery. WIREs Comput Mol Sci.

[6] Haider N, The checkmol/matchmol homepage. http://merian.pch.univie.ac.at/~nhaider/cheminf/cmmm.html

[7] Salmina ES, Haider N, Tetko IV (2016). Extended functional groups (EFG): an efficient set for chemical characterization and structure-activity relationship studies of chemical compounds. Molecules.

[8] Sterling T, Irwin JJ (2015). ZINC 15 – Ligand discovery for everyone. J Chem Inf Model.

[9] Baell JB, Holloway GA (2010). New substructure filters for removal of pan assay interference compounds (PAINS) from screening libraries and for their exclusion in bioassays J Med. Chem.

[10] Bruns RF (2012). Watson IA rules for identifying potentially reactive or promiscuous compounds. J Med Chem.

[11] Yang C, Tarkhov A, Marusczyk J, Bienfait B, Gasteiger J, Kleinoeder T, Magdziarz T, Sacher O, Schwab CH, Schwoebel J (2015). New publicly available chemical query language, CSRML, to support chemotype representations for application to data mining and modeling. J Chem Inf Model.

[12] Ertl P, Jelfs S, Muehlbacher J, Schuffenhauer A, Selzer P (2006). Quest for the rings - in silico exploration of ring universe to identify novel bioactive heteroaromatic scaffolds. J Med Chem.

[13] Gaulton A, Bellis LJ, Bento AP, Chambers J, Davies M, Hersey A, Light Y, McGlinchey S, Michalovich D, Al-Lazikani B, Overington JP (2012). ChEMBL: a large-scale bioactivity database for drug discovery. Nucleic Acids Res.

[14] Ertl P (2003). Cheminformatics analysis of organic substituents: identification of the most common substituents, calculation of substituent properties and automatic identification of drug-like bioisosteric groups. J Chem Inf Comp.

[15] Ertl P, Rohde B (2012). The molecule cloud - compact visualization of large collections of molecules. J Cheminform.

